# Concurrent validity of GLIM criteria in nutritional assessment of surgical patients with colorectal Cancer

**DOI:** 10.3389/fnut.2025.1691041

**Published:** 2026-01-02

**Authors:** Marina Bobos, Predrag Stevanovic, Rastko Zivic, Nemanja Dimic, Marko Djuric, Irina Nenadic, Milica Mijovic, Andrijana Vasic, Miodrag Lalosevic, Katarina Borocki, Vesna Stevanovic, Slavica Mutavdzin Krneta

**Affiliations:** 1 University Hospital Center Dr Dragisa Misovic, Belgrade, Serbia; 2 The Institute for Health Care for Mother and Child os Serbia dr. Vukan Cupic, Belgrade, Serbia; 3 Institute of Medical Physiology Richard Burijan, Belgrade, Serbia

**Keywords:** GLIM criteria, subjective global assessment, colorectal cancer, surgical patients, malnutrition, nutritional assessment, concurrent validity, perioperative nutrition

## Abstract

**Introduction:**

Malnutrition affects outcomes in surgical treatment of patients with colorectal cancer (CRC). The Global Leadership Initiative on Malnutrition (GLIM) proposed a framework of diagnostic criteria, but it needs validation in different settings. The aim of this study was to evaluate the concurrent validity of GLIM criteria—across all 21 phenotypic–etiologic combinations—against a standard (modified Subjective Global Assessment, mSGA) in surgical patients with CRC.

**Methods:**

Prospective cohort of 105 adults scheduled for elective CRC surgery (June 2023–June 2024). Nutritional risk was screened with Nutritional Risk Screening 2002 (NRS-2002). Nutritional status was assessed by mSGA and by GLIM across all 21 combinations. Agreement and classification differences were tested using McNemar’s test and Cohen’s Kappa; sensitivity and specificity of GLIM (vs mSGA) were calculated. Bootstrap procedures supported inference.

**Results:**

Overall, GLIM classified 55.2% of patients as malnourished vs. 31.4% by mSGA. McNemar’s test indicated a significant difference in classification (*p* < 0.001), while Kappa showed moderate agreement (*κ* = 0.505; 95% CI 0.368–0.656). Nine GLIM combinations did not differ significantly from mSGA by McNemar’s test; among these, four combinations - P1EA, P12EA, P13EA, and P123EA - demonstrated significant agreement with mSGA (highest concordance). Across combinations, malnutrition prevalence ranged from 13.3 to 55.2%. Using aggregate GLIM, sensitivity was 96.97% and specificity 63.89% versus mSGA; the best single combination (P12EA - unintentional weight loss, low BMI, and reduced food intake or assimilation) yielded sensitivity 84.85% and specificity 83.33%.

**Discussion:**

In elective CRC surgery, several GLIM combinations— particularly P1EA, P12EA, P13EA, and P123EA —show meaningful agreement with mSGA and may offer a more straightforward, pragmatic approach to identify malnutrition preoperatively. Adoption of these combinations could streamline assessment and support timely perioperative nutritional interventions. Larger multicenter studies should confirm these findings and determine predictive value for clinical outcomes.

## 1 Introduction

CRC is a malignant tumor of the large intestine. It is the third most common cancer worldwide, and the second most common cause of cancer mortality (1). Patients undergoing colorectal surgery are at risk of prolonged hospitalization, surgical site infections, and increased treatment costs - particularly among the elderly population (2). However, the length of hospital stay and the occurrence of postoperative complications are also significantly influenced by malnutrition, a state of inadequate nutritional status in patients (3). In a 2025 meta-analysis that included 35 studies and more than 9,000 colorectal cancer survivors, 33.13% of participants had moderate malnutrition, while 47.78% had some degree of malnutrition (4).

It has been clinically demonstrated that undernourished patients who received preoperative nutritional therapy had better surgical outcomes compared to those who did not (5). Unfortunately, in clinical practice, many cases of malnutrition remain unrecognized and untreated.

To date, there is no universally accepted gold standard for determining indications for initiating nutritional therapy (6). In clinical practice, three categories of data are used for this purpose: patient data obtained through anthropometric measurements, laboratory analyses of blood and urine samples, and findings from nutritional screening and assessment tools. All of these tools are complementary, and should be used in different combinations, depending on the target population and the clinical setting.

Given that the focus of this study is the evaluation of the concurrent validity of the GLIM criteria, anthropometric and laboratory parameters will not be further addressed. Instead, particular attention will be given to the epidemiological screening and assessment tools commonly used to evaluate nutritional status in surgical patients with cancer.

Among the screening tools applied in clinical practice, the most commonly used for surgical patients include NRS-2002, the Malnutrition Universal Screening Tool (MUST), and the Perioperative Nutrition Screen (PONS) (7–10). Once patients at nutritional risk are identified through screening, it is necessary to apply an appropriate tool for the assessment of nutritional status and determination of the degree of malnutrition. For this purpose, the Subjective Global Assessment (SGA) and its modified version, Patient-Generated Subjective Global Assessment (PG-SGA), are most commonly used (11–13).

NRS-2002 is a nutritional risk screening tool that is most commonly recommended and used in clinical practice (7, 8). It is simple to administer, and one of its main advantages is its applicability across a wide range of hospitalized patients. A limitation of this tool is that several of its components rely on subjective assessment.

The SGA is based on a clinical – yet subjective – evaluation of dietary intake, weight loss, and physical condition, using both subjective and objective patient information gathered through medical history and physical examination (11). It is considered a comprehensive tool that does not require laboratory testing or specialized equipment. However, it is often recommended that the SGA be performed independently by two evaluators on the same patient to ensure the reliability of the results. For this reason, its proper use and interpretation require specific training of healthcare professionals (12). There are also quantitative adaptations of the SGA, such as mSGA, that assign numerical scores to patient assessment findings, thereby classifying nutritional status into several categories (14).

Given the large number and diversity of existing nutritional screening and assessment tools, it is evident that implementing each of them for all patients in routine clinical practice would require substantial effort and resources. For this reason, there has been a long-standing search for a unified, simple, and practical solution that could be routinely applied across all patient populations. In response to this need, the Global Leadership Initiative on Malnutrition proposed a set of standardized criteria aimed at improving the identification and diagnosis of malnutrition in diverse clinical settings. As a result of expert consensus, the GLIM criteria were introduced in 2019 as a standardized approach to diagnosing malnutrition (15). These criteria consist of three phenotypic and two etiologic components, and a diagnosis of malnutrition requires the presence of at least one criterion from each group. An overview of the phenotypic and etiologic criteria, along with all potential positive combinations required to assume the presence of malnutrition, is presented in Table 1.

**Table 1:** Overview of all positive combinations of GLIM phenotypic and etiologic criteria.

Combination	Phenotypic criteria			Etiologic criteria		Combination code used in this paper
	Unintentional weight loss (P1)	Low BMI (P2)	Reduced muscle mass (P3)	Reduced food intake or assimilation (EA)	Inflammation (EB)	
1						P1EA
2						P1EB
3						P2EA
4						P2EB
5						P3EA
6						P3EB
7						P12EA
8						P12EB
9						P13EA
10						P13EB
11						P23EA
12						P23EB
13						P12EAB
14						P13EAB
15						P23EAB
16						P123EAB
17						P123EA
18						P123EB
19						P1EAB
20						P2EAB
21						P3EAB

Blue color for Unintentional weight loss (P1 criterion); Green color for Low Body Mass Index (P2 criterion); Yellow color for Reduced muscle mass (P3 criterion)/; Orange color for educed food intake or assimilation (EA criterion); Red color for Inflammation (EB criterion).

Since the GLIM criteria were developed based on expert knowledge, clinical opinion, and assumptions, there is a clear need to evaluate their validity across different combinations, populations, and patient groups, using both retrospective and prospective study designs.

This study aimed to assess the concurrent validity of the GLIM criteria by comparing them with mSGA – in evaluating nutritional status in surgical patients with colorectal cancer.

## 2 Materials and methods

This research was a prospective cohort study, which included 105 hospitalized patients at the Clinical.

University Hospital Center “Dr. Dragisa Misovic - Dedinje” scheduled for elective colorectal cancer surgery between June 1, 2023, and June 1, 2024.

Inclusion criteria were age of patients over 18 years with a confirmed diagnosis of a primary colorectal tumor, admitted for elective surgical treatment during the defined study period.

Exclusion criteria were emergency surgery and a history of prior surgical interventions involving the colorectal segment of the gastrointestinal tract, excluding polypectomy and temporary diverting colostomy performed as part of a planned surgical management. Additionally, patients who were unable to independently complete the preoperative and postoperative questionnaires due to mental illness and/or impaired consciousness were excluded from the study.

The study was approved by the Institutional Ethics Committee and registered at ClinicalTrials.gov (NCT05957744).

Nutritional status was evaluated using a comprehensive set of tools and parameters. The assessment included:

- Anthropometric measurements, such as body height, body weight, waist, hip, and upper arm circumference, triceps skinfold thickness, and calculation of BMI;
- NRS-2002 and mSGA as screening and assessment tools for nutritional status
- Malnutrition assessment using the GLIM criteria, applied across all 21 possible combinations of phenotypic and etiologic components.

Two items in the mSGA (decreased fat stores or loss of subcutaneous fat, and signs of muscle wasting) proved particularly challenging. For the assessment of the former, anthropometric measurement of triceps skinfold thickness was used, along with the percentage of body fat estimated through body composition analysis using a specialized scale (Tanita BC 601, Japan). Reference values for percentage of body fat and muscle mass percentage were adopted from a population-based British study by Dodds et al. (16) Muscle tissue loss was assessed not only by muscle mass percentage obtained from the scale, but also by evaluation of paravertebral muscle density. Paravertebral muscle density was measured on CT scans at the L3 level, specifically of the erector spine and multifidus muscles, and expressed in Hounsfield units (HU). The cut-off value was set at 50 HU. Values below this threshold were considered indicative of loss of muscle mass.

All patients underwent preoperative evaluation and were categorized into two groups – well-nourished and malnourished – based on both mSGA and GLIM criteria. Subsequently, results obtained using GLIM were compared with those from mSGA, both in general and across all 21 GLIM combinations individually, to examine consistency and agreement between the two approaches.

The Kolmogorov–Smirnov test was used to assess the normality of data distribution. The McNemar test and the Kappa coefficient were applied to determine the statistical significance of the findings, with a significance level set at *p* < 0.05. All analyses were performed using IBM SPSS Statistics, version 27.0.

## 3 Results

Table 2 shows the basic characteristics of the study participants.

**Table 2:** Baseline characteristics of the study population.

Variable	Mean ± SD / *n* (%)
Age (years)	66.3 ± 12.1
Sex (male)	64 (60.4%)
Charlson Comorbidity Index	5.23 ± 2.69 (min 0, max 12)
Body Mass Index (kg/m^2^)	25.5 ± 4.6
<18.5	4 (3.8%)
18.5–24.9	52 (49.5%)
25–29.9	31 (29.5%)
>30	18 (17.2%)
Primary Tumor Location	
Cecum	15 (14.3%)
Ascending colon	8 (7.6%)
Hepatic flexure	6 (5.7%)
Transverse colon	5 (4.8%)
Splenic flexure	4 (3.8%)
Descending colon	6 (5.7%)
Sigmoid colon	30 (28.6%)
Rectosigmoid junction	5 (4.8%)
Rectum	26 (24.7%)
Stage of Disease	
I	37 (35.2%)
IIa	15 (14.3%)
IIb	1 (0.9%)
IIc	2 (1.9%)
IIIa	2 (1.9%)
IIIb	17 (16.2%)
IIIc	14 (13.3%)
IVa	9 (8.6%)
IVb	8 (7.6%)
Preoperative treatment	
None	95 (90.5%)
Chemotherapy	8 (7.6%)
Chemotherapy and Radiotherapy	2 (1.9%)

Comparison of nutritional status according to mSGA and GLIM criteria was assessed using a 2 × 2 cross-tabulation (Table 3), McNemar’s test, and Cohen’s Kappa coefficient. The GLIM criteria demonstrated a sensitivity of 96.97% and a specificity of 63.89% when compared to the mSGA as a reference method.

**Table 3:** Cross-tabulation of nutritional status according to mSGA and GLIM criteria.

GLIM-based nutritional status			mSGA-based nutritional status		Total
			Well-nourished	Malnourished	
GLIM-based nutritional status	Well-nourished	Count	46	1	47
		% of Total	43.8%	1.0%	44.8%
	Malnourished	Count	26	32	58
		% of Total	24.8%	30.5%	55.2%
Total		Count	72	33	105
		% of Total	68.6%	31.4%	100.0%

McNemar’s test revealed a statistically significant difference in classification (*p* < 0.001), indicating that the two tools are not entirely interchangeable. Despite this, Cohen’s Kappa was 0.505 (*p* = 0.000), which means a moderate level of agreement between the two methods beyond chance.

The 95% confidence interval for Kappa ranged from 0.368 to 0.656, confirming the statistical reliability of the observed agreement. This was further supported by bootstrap analysis, which yielded consistent results.

Following the overall comparison between mSGA and the GLIM criteria, each specific combination of GLIM criteria (from Table 1) was subsequently compared with mSGA. The nutritional status assessment based on mSGA and the following nine combinations of GLIM criteria revealed no statistically significant differences:

- P1EA - unintentional weight loss and reduced food intake or assimilation (McNemar test *p =* 0.238, Kappa = 0.621, *p =* 0.000),
- P3EB - reduced muscle mass and disease burden or inflammation (McNemar test *p =* 0.052, Kappa = 0.470, *p =* 0.000),
- P12EA - unintentional weight loss, low BMI, and reduced food intake or assimilation (McNemar test *p =* 0.143, Kappa = 0.645, *p =* 0.000),
- P13EA - unintentional weight loss, reduced muscle mass, and reduced food intake or assimilation (McNemar test *p =* 0.064, Kappa = 0.609, *p =* 0.000),
- P23EA - low BMI, reduced muscle mass, and reduced food intake or assimilation (McNemar test *p =* 0.115, Kappa = 0.527, *p =* 0.000),
- P23EB - low BMI, reduced muscle mass, and disease burden or inflammation (McNemar test *p =* 0.678, Kappa = 0.479, *p =* 0.000),
- P23EAB - low BMI, reduced muscle mass, reduced food intake or assimilation, and disease burden or inflammation (McNemar test *p =* 0.832, Kappa = 0.506, *p =* 0.000),
- P123EA - unintentional weight loss, low BMI, reduced muscle mass, and reduced food intake or assimilation (McNemar test *p =* 0.064, Kappa = 0.609, *p =* 0.000), and
- P3EAB - reduced muscle mass, reduced food intake or assimilation, and disease burden or inflammation (McNemar test *p =* 0.052, Kappa = 0.470, *p =* 0.000).

However, among these nine combinations, only four demonstrated significant agreement with the mSGA assessment, namely: P1EA, P12EA, P13EA, and P123EA. This may indicate that these combinations of GLIM criteria could be used for the assessment of nutritional status as an alternative to the considerably more complex evaluation using mSGA. It appears that, for the assessment of nutritional status in surgical patients with colorectal cancer, the most important GLIM criteria are unintentional weight loss and reduced food intake or assimilation, as these two parameters are present in all four combinations that demonstrated significant agreement with mSGA.

## 4 Discussion

The development of colorectal cancer (CRC) is strongly influenced by dietary patterns. In 2024, an Italian research group developed a new tool - the Chrono Med Diet Score (CMDS) - and demonstrated that it can be used to assess the risk of developing gastrointestinal tumors, including CRC (17). Furthermore, a retrospective study showed that a modern dietary pattern leading to fatty liver disease is associated with an increased risk of colorectal cancer (18). In our study, patients completed a diary of food intake; however, it was used not for dietary pattern assessment, but for evaluating preoperative caloric intake as a part of the nutritional status assessment.

The GLIM Consortium emphasized the necessity of validating the GLIM criteria for the assessment of malnutrition in different settings, and in 2020 issued guidelines for each type of validation, highlighting criterion validity (including concurrent and predictive validity) as the most important (6). In the context of concurrent validity, the application of the GLIM criteria requires simultaneous comparison with the results of a recognized reference method. Following nutritional screening with the NRS-2002, it was decided to use the mSGA as the standard method.

In our study, the McNemar test demonstrated that the results of malnutrition assessment according to mSGA and GLIM criteria differed significantly. In contrast, the Kappa statistic indicated a moderate level of agreement between the two methods, which prompted further investigation and the evaluation of individual GLIM combinations.

The main finding of this study is that the results of malnutrition assessment using nine different combinations of GLIM criteria showed a certain degree of concordance with mSGA, with four combinations in particular (P1EA, P12EA, P13EA, and P123EA) demonstrating significant concordance with mSGA, which may suggest that the GLIM criteria could simplify the identification of malnutrition in these patients, which can optimize the patients’ preoperative condition and potentially reduce postoperative complications.

An observational retrospective study involving 885 oncology patients with head and neck tumors assessed nutritional status in patients at nutritional risk (determined by NRS-2002) using 14 different combinations of GLIM criteria, and then compared the results with those obtained by SGA (19). According to the SGA, 173 (26.1%) patients were malnourished (SGA categories B or C), while the prevalence of malnutrition according to the GLIM combinations ranged from 3.9 to 30.0%. Adequate concurrent and predictive validity were defined as sensitivity and specificity values >80% and odds ratio values ≥2.0, respectively. None of the tested combinations reached adequate concurrent validity; however, malnutrition defined by four combinations independently predicted surgical complications. In contrast to this study, ours was prospective in design, which allowed the inclusion of additional parameters in the assessment. Instead of the standard SGA, our study used a modified version in order to minimize subjective evaluation as much as possible. Moreover, our study focused on patients with colorectal cancer and examined all 21 combinations of GLIM criteria. The McNemar test identified nine combinations whose results did not differ significantly from those of mSGA. As many as four combinations demonstrated significant agreement with mSGA, as confirmed by the Kappa statistic.

A retrospective study from 2022 on a group of 918 patients with colorectal cancer showed that malnourished patients (according to the GLIM criteria) experienced more frequent postoperative complications and poorer treatment outcomes compared to well-nourished patients (20). Although this study examined the predictive validity of the GLIM criteria, it shares many similarities with our own. This further highlights the potential importance of using the simple GLIM criteria for the identification of malnutrition.

One prospective study conducted in 2020 on 206 hospitalized surgical patients examined the practical applicability and validity of the GLIM framework in comparison with the SGA (21). Each combination of criteria demonstrated a different prevalence of malnutrition, ranging from 10.7 to 41.3%, whereas according to the SGA, half of the patients were classified as malnourished. In our study, although conducted on nearly half the number of participants, a higher proportion of patients were classified as malnourished when using the GLIM framework compared to the results obtained with mSGA (55.2 and 31.4%, respectively). Different combinations of GLIM criteria showed varying prevalence rates of malnutrition, ranging from 13.3 to 55.2%. These findings are consistent with previous studies that reported wide variability depending on the specific GLIM combinations applied.

In another, larger study involving 1,115 oncology patients preparing for major abdominal surgery, nutritional status was evaluated using both the SGA and the GLIM criteria (22). Prior to this, nutritional risk screening had been performed using several different epidemiological questionnaires. The accuracy of malnutrition diagnosis according to the GLIM criteria compared with the SGA varied depending on which screening test had been previously applied, with the highest reliability of the GLIM criteria observed with the Mini Nutritional Assessment Short Form (MNA-SF, AUC 0.78).

In our study, NRS-2002 was used as the screening tool. Our results might have differed had another screening tool, such as the MNA-SF, been applied. This illustrates the importance of selecting the initial screening instrument because it influences the diagnostic performance of the GLIM criteria and the comparability of results across studies. Nevertheless, our findings demonstrate that the GLIM framework has considerable potential for identifying malnutrition in surgical patients with CRC.

A study from Brazil compared hospitalized patients and found that 33.9% of them were malnourished according to SGA, but when the GLIM framework was used, 41.6% were classified as malnourished (23). The GLIM criteria demonstrated satisfactory accuracy (AUC = 0.842; 95% CI: 0.807–0.877), with a sensitivity of 86.6% and a specificity of 81.6%. This study included all hospitalized patients except those admitted as emergencies or those treated in intensive care units. The main difference from our study lies in the patient population. Although our study also excluded patients from ICUs and those undergoing emergency procedures, it was focused exclusively on surgical patients with colorectal cancer. These patients often experience digestive disturbances, some undergo bowel preparation prior to surgery, and many report reduced food intake in their dietary records. Similarly to the Brazilian study, a higher proportion of patients in our cohort were classified as malnourished by the GLIM criteria than by mSGA. For the overall GLIM framework, sensitivity was 96.97% and specificity was 63.89%, while the best-performing combination (P12EA) showed sensitivity of 84.85% and specificity of 83.33%. Differences in study populations and methodological approaches may partly explain the variation in diagnostic performance between studies.

The GLIM criteria are considerably simpler than previously available tools for nutritional screening and assessment, representing a potentially practical and straightforward solution for identifying patients who could benefit from nutritional therapy. This is particularly important in oncology patients preparing for surgery. The findings of this study suggest that, for this patient population, the GLIM combinations P1EA, P12EA, P13EA, and P123EA are especially relevant, as they demonstrated the highest concordance with mSGA results. In this way, the time required for nutritional status assessment could be significantly reduced, allowing for more efficient planning of perioperative nutritional therapy and support in surgical patients with colorectal cancer.

This study has several limitations. First, the sample size was relatively small (105 patients), which limits the statistical power and generalizability of the findings. Second, the study was conducted in a single center, which may reduce the external validity and applicability of the results to other clinical settings. Third, the results cannot be extrapolated to oncological or surgical patients other than those with CRC. Fourth, although the mSGA was used as a standard in order to minimize subjectivity, it is still not a universally accepted standard for nutritional assessment. In addition, some methodological limitations should be acknowledged: body composition analysis was performed using bioelectrical impedance, which has lower accuracy compared to methods such as DEXA; and CT-based muscle mass assessment was limited to patients with available imaging of sufficient quality.

In conclusion, our study demonstrated that among surgical patients with CRC, nine different combinations of GLIM criteria showed concordance with mSGA, with four combinations (P1EA, P12EA, P13EA, and P123EA) of considerable agreement. This means that the GLIM framework, particularly the above-mentioned combinations, may serve as a more straightforward and more practical alternative to mSGA for the identification of malnutrition in patients with CRC. With faster and less complex assessment of nutritional status, the GLIM criteria could optimize clinical approach to patients with preoperative malnutrition and improve the planning of perioperative nutritional support. Future studies should be multicenter and with larger cohorts to validate these findings and to explore the predictive value of the GLIM criteria for clinical outcomes such as postoperative complications, length of hospital stay, and survival.

## Data Availability

The data analyzed in this study is subject to the following licenses/restrictions: data supporting the findings of this study are available from the corresponding author upon reasonable request. Requests to access these datasets should be directed to MB, marinamorisan@gmail.com.
