# Supplementary material for: Concurrent validity of GLIM criteria in nutritional assessment of surgical patients with colorectal Cancer
Source: Front Nutr. 2026 Jan 2;12:1691041. doi: 10.3389/fnut.2025.1691041 (PMC12807904; doi:10.3389/fnut.2025.1691041)
Supplement: Supplementary file 1 [file Table_1.docx]

|  | **Phenotypic criteria** | | | **Etiologic criteria** | | **Combination code used in this paper** |
| --- | --- | --- | --- | --- | --- | --- |
| **Combination** | Unintentional weight loss  (P1) | Low  BMI  (P2) | Reduced musscle mass  (P3) | Reduced food intake or  assimilation  (EA) | Inflamation  (EB) |  |
| 1 |  |  |  |  |  | P1EA |
| 2 |  |  |  |  |  | P1EB |
| 3 |  |  |  |  |  | P2EA |
| 4 |  |  |  |  |  | P2EB |
| 5 |  |  |  |  |  | P3EA |
| 6 |  |  |  |  |  | P3EB |
| 7 |  |  |  |  |  | P12EA |
| 8 |  |  |  |  |  | P12EB |
| 9 |  |  |  |  |  | P13EA |
| 10 |  |  |  |  |  | P13EB |
| 11 |  |  |  |  |  | P23EA |
| 12 |  |  |  |  |  | P23EB |
| 13 |  |  |  |  |  | P12EAB |
| 14 |  |  |  |  |  | P13EAB |
| 15 |  |  |  |  |  | P23EAB |
| 16 |  |  |  |  |  | P123EAB |
| 17 |  |  |  |  |  | P123EA |
| 18 |  |  |  |  |  | P123EB |
| 19 |  |  |  |  |  | P1EAB |
| 20 |  |  |  |  |  | P2EAB |
| 21 |  |  |  |  |  | P3EAB |
